# Diversity of the Bacterial and Fungal Microflora from the Midgut and Cuticle of Phlebotomine Sand Flies Collected in North-Western Iran

**DOI:** 10.1371/journal.pone.0050259

**Published:** 2012-11-30

**Authors:** Mohammad Akhoundi, Rounak Bakhtiari, Thomas Guillard, Ahmad Baghaei, Reza Tolouei, Denis Sereno, Dominique Toubas, Jérôme Depaquit, Mehdi Razzaghi Abyaneh

**Affiliations:** 1 Université de Reims Champagne-Ardenne, ANSES, EA4688 - USC, Transmission Vectorielle et Epidémiosurveillance de Maladies Parasitaires (VECPAR), Université de Reims Champagne-Ardenne, Faculté de Pharmacie, Reims, France; 2 Mycology and Parasitology Department, Pasteur Institute of Iran, Tehran, Iran; 3 Department of Microbiology, School of Public Health and Institute Health Research, Tehran University of Medical Sciences, Tehran, Iran; 4 UFR Médecine, SFR CAP-Santé (FED 4231), Université de Reims Champagne-Ardenne, Reims, France; 5 MIVEGEC, UMR IRD 224-CNRS 5290-UM1-UM2, Montpellier, France; 6 Unité MEDyC, FRE 3481 URCA CNRS, Université de Reims Champagne-Ardenne, Reims, France; 7 Laboratoire de Bactériologie, CHU de Reims, Hôpital Robert Debré, Reims, France; 8 Laboratoire de Parasitologie Mycologie, CHU de Reims, Hôpital Maison Blanche, Reims, France; Wadsworth Center, United States of America

## Abstract

**Background:**

Phlebotomine sand flies are the vectors of the leishmaniases, parasitic diseases caused by *Leishmania* spp. Little is known about the prevalence and diversity of sand fly microflora colonizing the midgut or the cuticle. Particularly, there is little information on the fungal diversity. This information is important for development of vector control strategies.

**Methodology/Principal Findings:**

Five sand fly species: *Phlebotomus papatasi, P. sergenti, P. kandelakii, P. perfiliewi* and *P. halepensis* were caught in Bileh Savar and Kaleybar in North-Western Iran that are located in endemic foci of visceral leishmaniasis. A total of 35 specimens were processed. Bacterial and fungal strains were identified by routine microbiological methods. We characterized 39 fungal isolates from the cuticle and/or the midgut. They belong to six different genera including *Penicillium* (17 isolates), *Aspergillus* (14), *Acremonium* (5), *Fusarium* (1), *Geotrichum* (1) and *Candida* (1). We identified 33 Gram-negative bacteria: *Serratia marcescens* (9 isolates), *Enterobacter cloacae* (6), *Pseudomonas fluorescens* (6), *Klebsiella ozaenae* (4), *Acinetobacter sp.* (3), *Escherichia coli* (3), *Asaia sp.* (1) and *Pantoea sp.* (1) as well as Gram-positive bacteria *Bacillus subtilis* (5) and *Micrococcus luteus* (5) in 10 isolates.

**Conclusion/Significance:**

Our study provides new data on the microbiotic diversity of field-collected sand flies and for the first time, evidence of the presence of *Asaia* sp. in sand flies. We have also found a link between physiological stages (unfed, fresh fed, semi gravid and gravid) of sand flies and number of bacteria that they carry. Interestingly *Pantoea* sp. and *Klebsiella ozaenae* have been isolated in Old World sand fly species. The presence of latter species on sand fly cuticle and in the female midgut suggests a role for this arthropod in dissemination of these pathogenic bacteria in endemic areas. Further experiments are required to clearly delineate the vectorial role (passive or active) of sand flies.

## Introduction

Phlebotomine sand flies are the natural exclusive vectors of leishmaniases, a group of parasitic diseases caused by protozoan kinetoplastid flagellates belonging to the genus *Leishmania*. They affect about 12 million people in many countries located in Mediterranean, tropical and sub-tropical regions [1], [2].

Sand flies harbor a huge variety of microorganisms, and not all are resident in the gut [3].They can also originate from the sand flies’ external environment. In nature, adult Phlebotomine sand flies can colonize highly divergent environments e.g. tropical forests, temperate regions and deserts, in wild, domestic or anthropized biotopes. Adult sand flies usually remain close (less than one kilometer) from their larval development sites [4]. The sites where larval development take place are usually a mixture of animal faeces and mud which are found in both wild (rodent burrows, forest floors, caves) and anthropized biotopes (villages, animal shelters) [5], [6]. Larvae feed on the decomposing organic materials in these sites and the adults can therefore acquire a part of their microflora during their larval development. Furthermore, male and female sand flies feed daily on natural sugars, especially nectars or sap secretions and drink water from plants [7]. These sugars are the main source of carbohydrates for adults. Additionally, females require a blood-meal to complement their diet, during the maturation of their eggs and completion of the gonotrophic cycle [8], [9], [10]. During these feeding events, they can also acquire various microorganisms including bacteria (e.g. *Bartonella bacilliformis)*, fungi, Phleboviruses or other trypanosomatidae and co-colonization by human pathogenic and non pathogenic species of *Leishmania* i.e *L. turanica*, *L. gerbilli* and *L. major* [11], [12]. Beside the lumen of the gut, the diversity of microorganism present on the cuticle that is acquired independently of their breeding, feeding and resting places might also be highly informative for sand fly biology.

The majority of studies dealing with the microflora that colonizes gut of hematophagous insects were performed on mosquitoes [13]. However, very little is known about sand fly microflora and its’ possible impact on the biology (including longevity), reproduction and sand fly-pathogen interaction. This information is important for the development of new strategies for vector control. To date, a few investigations focused on the midgut bacterial flora have been carried out on *Lutzomyia longipalpis*, *Phlebotomus papatasi*, *P. tobbi*, *P. argentipes*, *P. duboscqi* and *Sergentomyia* spp. only [14], [15], [16], [17], [18], [19], [20].

Visceral leishmaniasis or Kala-azar, is a life-threatening parasitic infection, caused by *L. infantum* in Iran. The two provinces of Azarbaijan-e-sharqi and Ardabil used in the present study are two out of four main endemic foci of VL in Iran. Two others including Fars and Bushehr provinces are located in the south of the country. Several investigations have been carried out on the population composition and *Leishmania* infection of sand flies in different localities within these provinces. *P. perfiliewi transcaucasicus* and *P. kandelakii* have been reported as the proven vectors of VL in Azarbaijan-e-sharqi and Ardabil [21], [22]. Parvizi et al. 2008 [23] and Sanei Dehkordi et al. 2011 [24] have reported *P. perfiliewi* as the proven vector of VL in Kaleybar and Bileh Savar counties.

The aim of our study was to investigate the diversity of the microbial flora including bacteria and fungi that colonize both the cuticle and midgut in wild populations of sand flies through a culture dependent methodology. Specimens of sand flies investigated were prevalent in the endemic foci of visceral leishmaniasis in North-Western Iran and are representative of the sand fly diversity in this area.

## Materials and Methods

### Study Area

Sampling was carried out in August 2011 from rural regions of Kaleybar (38°58′ N 47°13′ E) and Bileh Savar (39°03′ N 48°31′ E) in Azarbaijan Sharqi and Ardabil provinces. These provinces are well-known endemic foci of visceral leishmaniasis in North-Western Iran. Five CDC miniature light traps were used in each sampling site and placed in houses, animal shelters, yards and rodents’ burrows. All light traps were sterilized by ethanol full spraying just before use and installed before sunset and remained functional throughout the night until the next morning.

### Processing of Phlebotomine Sand Flies

**Figure 1:**
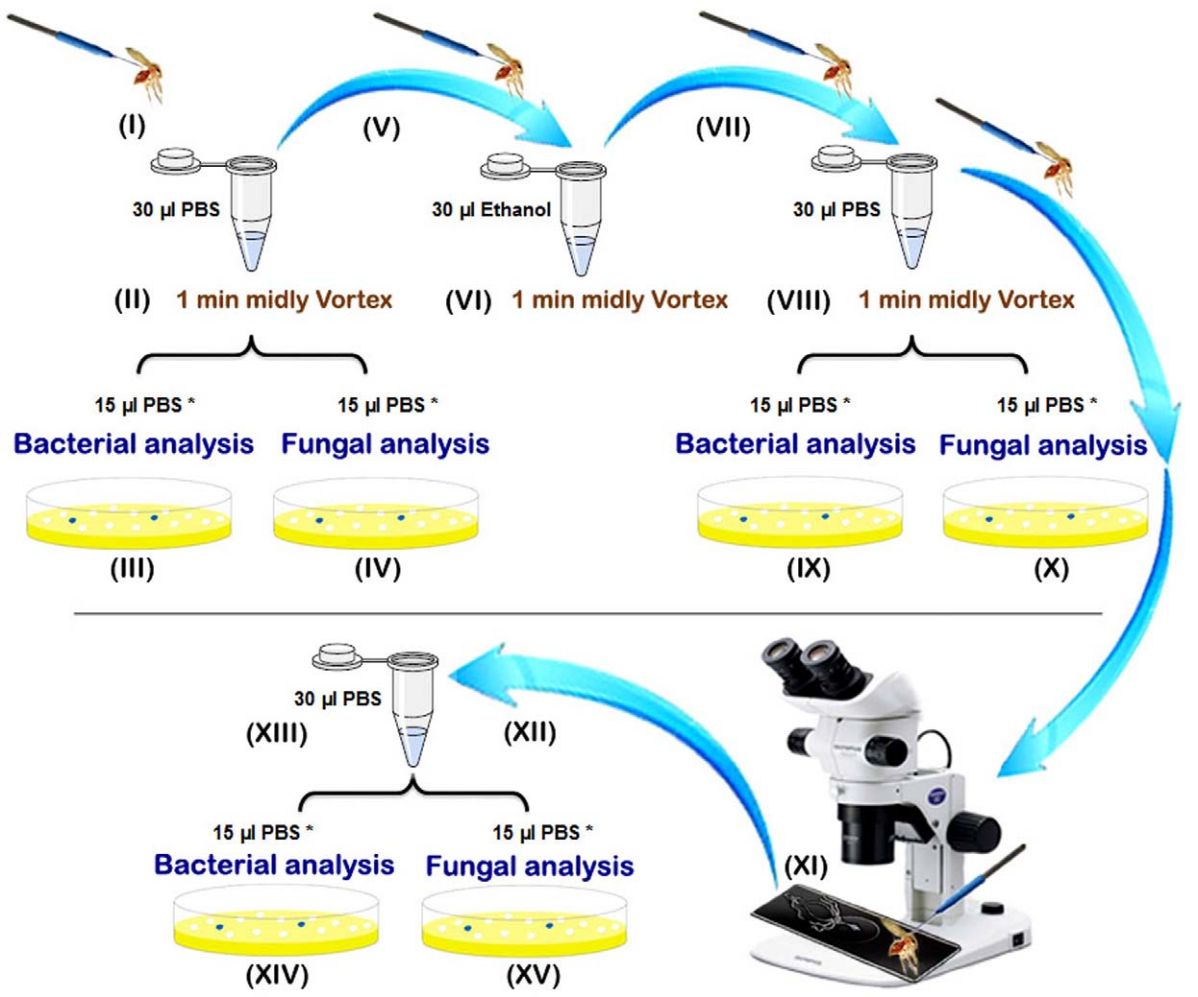
Preparation steps of the sand fly processing for fungal and bacterial analyses. *: include primary volume (15 µl) which were diluted up to 100 µl for microbial assessments.

Sand flies caught alive were processed individually using sterile single-use materials and reagents as summarized in the Figure 1. Sand flies were killed using cold shock (**I**) and males and females, subdivided in unfed, fresh fed (having red fresh blood non digested in their gut), semi gravid (having some eggs and a part of digested dark red to brown blood in their gut) and gravid specimens (with an abdomen full of eggs), were processed individually according the following protocol. Each individual sand fly was placed in a 1.5 ml microtube containing 30 µl PBS (Phosphate Buffered Saline). Samples were mildly vortex for 1 minute (**II**) and then 15 µl of the PBS was taken for bacterial isolation (**III**) and another 15 µl of the PBS for fungal characterization (**IV**) of the cuticle microflora. Each sand fly specimen was then transferred using sterile entomological micro needles to a new sterile microtube containing 30 µl absolute ethanol (**V**). After 1 min of mild vortex mixing (**VI**), the sand fly specimens were transferred to a new sterile microtube and washed with 30 µl of PBS (**VII**). After vortexing as previously described (**VIII**), 15 µl of the PBS was used for bacterial anlysis (**IX**) and 15 µl for fungal analysis (**X**) in order to check the sterilization of the cuticle. The sand flies were then removed from the microtubes and dissected under a stereomicrosope on a sterile microscopic slide with a drop of sterile PBS using sterile entomological micro needles. After the sand fly’s head was removed, the digestive tract was isolated on the microscopic slide (**XI**), washed with 30 µl PBS into a new sterile microtube (**XII**), and crushed using a glass pestle (**XIII**). Then, 15 µl of the PBS was used for bacterial analysis (**XIV**) and 15 µl for fungal analysis (**XV**) of the gut microflora.

### Mycological Identification

The PBS extracts from the cuticle and gut (**IV** and **XV**) were diluted with PBS to give a final volume of 100 µl and these diluted solutions were spread on to Sabouraud Dextrose Agar (Peptone 1%, Glucose 2%, Agar-agar 1.5%; Merck, Germany) and Potato Dextrose Agar (Potato infusion 20%, Dextrose 2%, Agar 2%) plates and incubated for 2 weeks at 25°C. The plates were periodically checked for fungal colonies. Identification of fungal isolates was performed according to a combination of macro- and microscopic morphology. Yeasts were identified by the use of the chromogenic medium Chromagar *Candida* ID2 (BioMérieux, France).

### Bacterial Identification

The 15 µl PBS solutions from steps **III** and **XIV** were also diluted to a final volume of 100 µl by the addition of PBS. Fifty µl of each were then used for a bacterial colony count assessment. To determine the number of colony forming units (CFU), the samples were serially diluted 10 times (from 1^1^ to 1^10^) and aliquots of 100 µl were transferred on the PCA: Plate Count Agar (105463, E. Merck Co.; Darmstadt, Germany). Plates were then incubated at 35°C for 48 h. Total colony counts were recorded for each dilution and the average for every sample was calculated.

The remaining 50 µl of each starting extract was then transferred into BHI broth (Brain Heart Infusion broth) medium and incubated at 37°C for 24 hours. Then, bacteria were plated on BHI agar, XLD (Xylose-Lysine-Desoxycholate), Hektoen enteric agar, MacConkey agar and blood agar media containing Amphotericin B (2 µg/ml) and incubated at 37°C for 24–48 hours.

The initial identification of bacterial species was based on the colony characteristics (involving colony size, shape, color, margin, opacity, elevation and consistency) and the morphology of isolates based on Gram’s staining procedure.

Finally, the API identification kit (API 20E, BioMerieux) was used for final identification of Gram-negative bacteria. The identification of Gram-positive bacteria was performed using the API Staph, API 20 Strep and API50CH B following the manufacturer’s recommendations.

### Statistical Analysis

Pearson Chi-Square and Scheffe’s tests based on the colony counting was performed using SPSS software ver. 18 to detect statistical differences in bacterial populations isolated from cuticle and midgut of (i) *P. papatasi* versus all other species using Pearson Chi-Square test and (ii) to compare males with unfed females, unfed females with freshly fed females, freshly fed females with semi-gravid females and semi-gravid females with gravid females using Scheffe’s test.

## Results

### Prevalence of Micro-organisms in Field Caught Sand Flies

**Figure 2:**
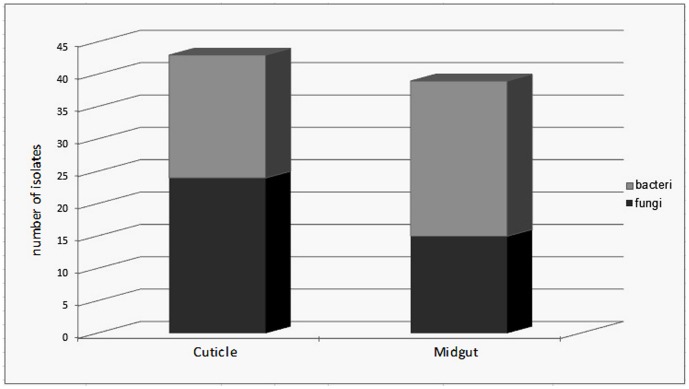
Mean number of bacteria and fungi isolated from cuticle or midgut of sand flies. These microorganisms were processed as described in the material and methods and the diversity of bacteria and fungi was ascertained by culture dependent methods.

**Figure 3:**
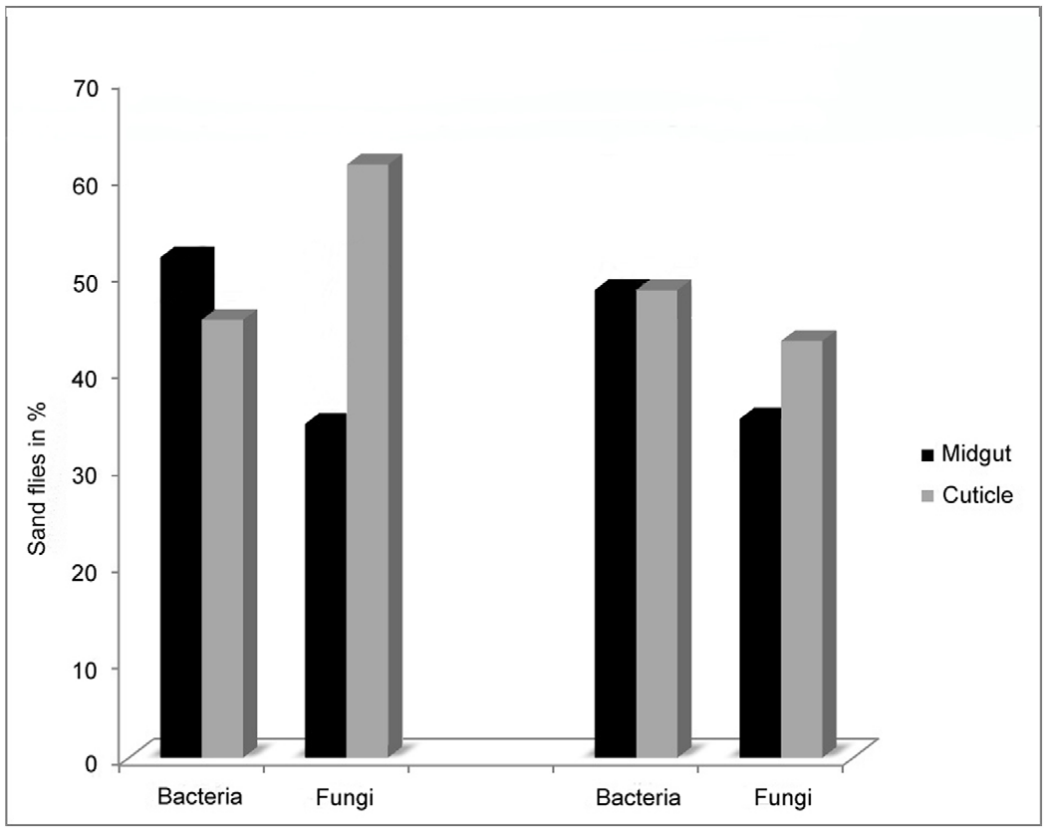
Frequency of bacterial or fungal isolation from the midgut or the cuticle of sand flies. *P. papatasi* appears on the left panel, and of the other species tested, on the right panel (i. e. *P. sergenti*, *P. perfiliewi*, *P. kandelakii* and *P. halepensis*).

**Figure 4:**
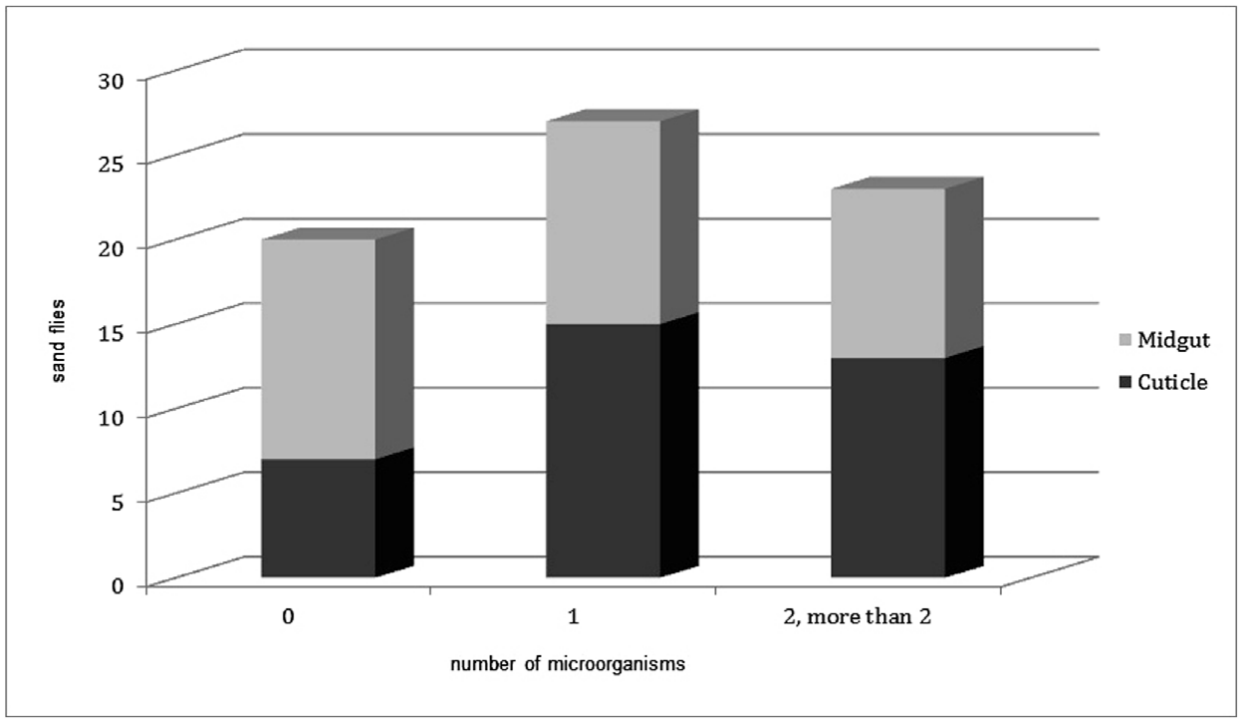
Mean number of sand flies carrying no, one or multiple microorganisms on their cuticle or midgut.

The aim of the study was to investigate the diversity of the bacterial and fungal strains that Phlebotomine sand flies carry with a culture dependent method. A total of 35 sand flies, 9 males and 26 females, belonging to five species (*P. papatasi. P. sergenti*, *P. kandelakii*, *P. perfiliewi* and *P. halepensis*) were collected. Sand flies were trapped in two different habitats, animal shelter (21) and outdoors (14). Samples were dissected and microorganisms were collected according to the procedure described in Figure 1. The sterilization efficiency was controlled during the whole procedure, see step VIII in Figure 1 and all samples correctly controlled (without any bacterial and fungal growing) for this step were included in the study and processed further. This methodology allowed us to readily isolate and identify the microorganisms present on the cuticle of the insect and those colonizing their midgut. Among the 35 processed sand flies, only 4 of them (3 males and 1 female) were negative for both bacteria and fungi on their cuticle or in their midgut. Among the 31 sand flies that bore microorganisms, slightly more microorganisms could be isolated from the cuticle than from their midgut. Interestingly, fungi were isolated more frequently on the cuticle than in the midgut and conversely bacteria were isolated more frequently from midgut than from cuticle (see Figure 2). Surprisingly, 37% of the midgut samples obtained from *P. papatasi* carried at least one fungal species whereas 63% of the cuticle samples originating from *P. papatasi* bore at least one fungal species. Such differences were not observed for the cuticle nor when looking at the presence of bacteria on the cuticle or in the sand fly midgut (see Figure 3). No differences were observed between males and females (data not shown), although this can not be statistically evaluated, because of the low number of male samples processed (9) as compared to females (26). The proportion of sand flies carrying no, one or multiple microorganisms (bacteria and/or fungi) in their midgut appears to be roughly the same (see Figure 4). By contrast, we observed that mono infection by bacteria and/or fungi is more frequent than no or multiple infection at the level of their cuticle. Finally, among the processed females, more bacterial strains are present on the cuticle and in the midgut of gravid compared to other physiological stages. The prevalence of bacteria increased progressively (except on the cuticle of semi-gravid females) with the advancement of the gonotrophic cycle (see Table 1).

**Table 1:** Synthesis of the average number of bacteria (plain) and fungal (bold) strains from the cuticle and the midgut of sand flies, according to sex, species and habitats.

Species	County	Habitat	No.	Cuticle				
				M	F			
					UnFed	Freshly Fed	Semi Gravid	Gravid
*P. papatasi*	Bileh Savar(Goon Papagh)	A.Sh	3		neg.**neg.**	neg. ***Penicillium*** ** sp.1**	*Pantoea* sp.(−)**neg.**	
		Outdoor	3	*Bacillus subtilis* (+)**neg.**		neg. ***Penicillium*** ** sp.1 ** ***Aspergillus terreus***		*Micrococcus luteus* (+) ***Aspergillus*** ** sp.**
	Bileh Savar(Gog Tapeh)	A.Sh	3		neg.***Penicillium*** ** sp.1**	*Serratia marcescens* (−) **neg**.		neg.***Aspergillus nidulans***
		Outdoor	3	*Bacillus subtilis* (+)***Fusarium*** ** sp.**	neg.**neg.**			*Klebsiella ozaenae* (−)**neg**.
	Kaleybar(Khane Khosro)	A.Sh	4		neg.***Penicillium*** ** sp.1** **A** ***spegillus fumigatus***	*Acinetobacter* sp. (−)*Klebsiella ozaenae* (−) **neg.**	neg. ***Penicillium*** ** sp.2**	*Pseudomonas fluorescens* (−) ***Penicillium*** ** sp.2**
		Outdoor	3	*Micrococcus luteus* (+) ***Acremonium*** ** sp.**		neg. ***Aspergillus flavus***	neg. ***Penicillium*** ** sp.1**	
*P. sergenti*	Bileh Savar(Goon Papagh)	A.Sh	1				*Pseudomonas fluorescens* (−)*Micrococcus luteus* (+) **neg**.	
		Outdoor	1			*Asaia* sp. (−) ***Penicillium*** ** sp.1 ** ***Aspergillus*** ** sp.**		
	Bileh Savar(Gog Tapeh)	A.Sh	1				neg. **neg.**	
	Kaleybar(Khane Khosro)	A.Sh	1	*Serratia marcescens* (−)***Aspergillus*** ** sp.** ***Geotrichum*** ** sp.**				
*P. kandelakii*	Kaleybar(Khane Khosro)	A.Sh	1		*Serratia marcescens* (−)**neg.**			
		Outdoor	1					neg.***Penicillium*** ** sp.1**
*P. perfiliewi*	Bileh Savar(Goon Papagh)	A.Sh	2	neg.**neg.**				*Serratia marcescens* (−) ***Penicillium*** ** sp.3**
	Bileh Savar(Gog Tapeh)	Outdoor	1				neg. ***Penicillium*** ** sp.1 ** ***Aspergillus flavus***	
	Kaleybar(Khane Khosro)	A.Sh	1	*Pseudomonas fluorescens* (−) **neg.**				
*P. halepensis*	Bileh Savar(Goon Papagh)	A.Sh	1	neg. ***Acremonium*** ** sp.**				
	Bileh Savar(Gog Tapeh)	Outdoor	1					neg.**neg**.
	Kaleybar(Khane Khosro)	A.Sh	3	neg.**neg.**	*Bacillus subtilis* (+)**neg.**		*Serratia marcescens* (−) ***Penicillium*** ** sp.3**	
		Outdoor	1	neg. **neg.**				
Total			**35**	0.56**0.44**	0.33**0.33**	0.50 **0.67**	0.43 **0.57**	0.57**0.71**

		Midgut							
Average number ofmicroorganisms(CFU^a^)		M	F				Average number ofmicroorganisms(CFUa)		
			UF	FF	SG	G			
2×10^4^			neg.**neg.**	neg.**neg.**	*Serratia marcescens* (−)*Bacillus subtilis* (+) ***Penicillium*** ** sp.1**		2×10^3^4×10^3^		
3×10^2^	2×10^4^	*Pseudomonas fluorescens*(−)**neg.**		*Bacillus subtilis* (+)*Pseudomonas fluorescens* (−)**neg**.		neg.***Aspergillus*** ** sp.**	1×10^2^	1×10^3^3×10^3^	
2×10^3^			neg.**neg.**	neg.***Acremonium*** ** sp.**		*Micrococcus luteus* (+)**neg.**	2×10^5^		
2×10^2^	2×10^4^	neg. ***Penicillium*** ** sp.1**	*Enterobacter cloacae* (−)**neg.**			neg.**neg.**	3×10^2^		
2×10^3^4×10^3^	3×10^4^		neg.**neg.**	*Escherichia coli* (−)*Enterobacter cloacae* (−)***Acremonium*** ** sp., ** ***Aspergillus*** **sp.**	*Enterobacter cloacae* (−) ***Penicillium*** ** sp.1** ***Acremonium*** ** sp.**	*Serratia marcescens* (−) ***Candida Albicans***	2×10^3^2×10^3^	3×10^4^	2×10^5^
3×10^2^		*Acinetobacter* sp. (−) **neg.**		*Klebsiella ozaenae* (−)**neg.**	neg.**neg.**		2×10^2^	1×10^3^	
3×10^4^2×10^4^					neg.**neg.**		neg.		
1×10^3^				neg.**neg.**			neg.		
neg.					*Acinetobacter* sp. (−) **neg.**		3×10^3^		
2×10^2^		*Enterobacter cloacae* (−) ***Aspergillus fumigatus***					2×10^2^		
3×10^2^			neg**. neg.**				neg.		
neg.						*Escherichia coli* (−)**neg.**	2×10^4^		
3×10^4^		neg.**neg.**				*Escherichia coli.* (−)*Enterobacter cloacae* (−) ***Panicillium*** ** sp.3**	2×10^4^2×10^5^		
neg.					*Enterobacter cloacae*(−) ***Aspergillus flavus***		3×10^3^		
4×10^2^		neg. ***Aspergillus*** ** sp.**					neg.		
neg.		*Serratia marcescens* (−) **neg.**					2×10^3^		
neg.						*Klebsiella ozaenae* (−)*Pseudomonas fluorescens* (−)***Panicillium*** ** sp.1**	2×10^4^3×10^3^		
4×10^2^	3×10^3^	neg.**neg.**	*Micrococcus luteus* (+)*Serratia marcescens* (−) ***Aspergillus flavus***		neg.**neg.**		1×10^3^2×10^3^		
neg.		neg. **neg.**					neg.		
		0.44**0.33**	0.33**0.17**	0.50**033**	0.57**0.43**	0.71**0.57**			

neg.: negative M: Male F: Female UF: UnFed FF: Freshly Fed SG: Semi Gravid G: Gravid A.sh: Animal shelter (+): Gram-positive bacteria (−): Gram-negative bacteria.

a Colony forming unit.

### Mean Number of Bacterial Colonies Per Individuals

The individual results are presented in Table 1. Because the number of sampled sand flies is not in equilibrium (include more *P. papatasi* than all other species), we have analyzed the data in two groups: *P. papatasi* (n = 19) and species belonging to another species (n = 16).

First of all, cuticle and midgut isolated from *P. papatasi* produced a significantly, lower number of colonies than the other species for cuticle (<chi>2 = 1592, *P*<0.05) and midgut (<chi>2 = 11500, *P*<0.05) respectively.

In addition, the average number of colonies obtained from cuticle of *P. papatasi* was higher in females than males and depended on the physiological state of the insects, the semi gravid females producing a higher number of colonies (2.6×10^2^ for the males and 2.6×10^3^ for the freshly fed females, 2×10^4^ for the semi-gravid females and 2.6×10^4^ for the gravid ones). The same trends were observed for the other species (mean = 3×10^2^ for the males, 3.5×10^2^ for the unfed females, 1×10^3^ for the freshly fed females, 1.7×10^4^ for the semi-gravid females and 3×10^4^ for the gravid ones). A similar observation performed on the midgut of *P. papatasi* showed that the numbers of colonies were: 1.5×10^2^ for the males, 3×10^2^ for the unfed females, 2.7×10^3^ for the freshly fed females, 1.8×10^4^ for the semi-gravid females and 2×10^5^ for the gravid ones. By comparison the colonies obtained for the other species were: 1.1×10^3^ for the males, 3×10^3^ for the unfed females, 3×10^3^ for the semi-gravid females and 8.7×10^4^ for the gravid ones. Differences in quantities of bacteria in males and the different physiological stages of females were also analyzed using Scheffe’s test. There was no significant differences in the numbers of colonies derived from the cuticle of *P. papatasi* and other groups for males-unfed females, unfed-freshly fed physiological stages (*P*>0.0125) whereas they were significant for freshly fed-semi gravid and semi gravid-gravid (*P*<0.0125).

There were no significant differences in bacterial colonies isolated from the midgut for males-unfed, unfed-freshly fed, freshly fed-semi gravid physiological stages (*P*>0.0125) and only it was significant between semi gravid and gravid females (*P*<0.0125).

It seems therefore that species, sex and physiology have some influences on the bacterial colonization of the cuticle and the midgut of sand flies.

### Diversity of Micro-organisms Isolated

**Figure 5:**
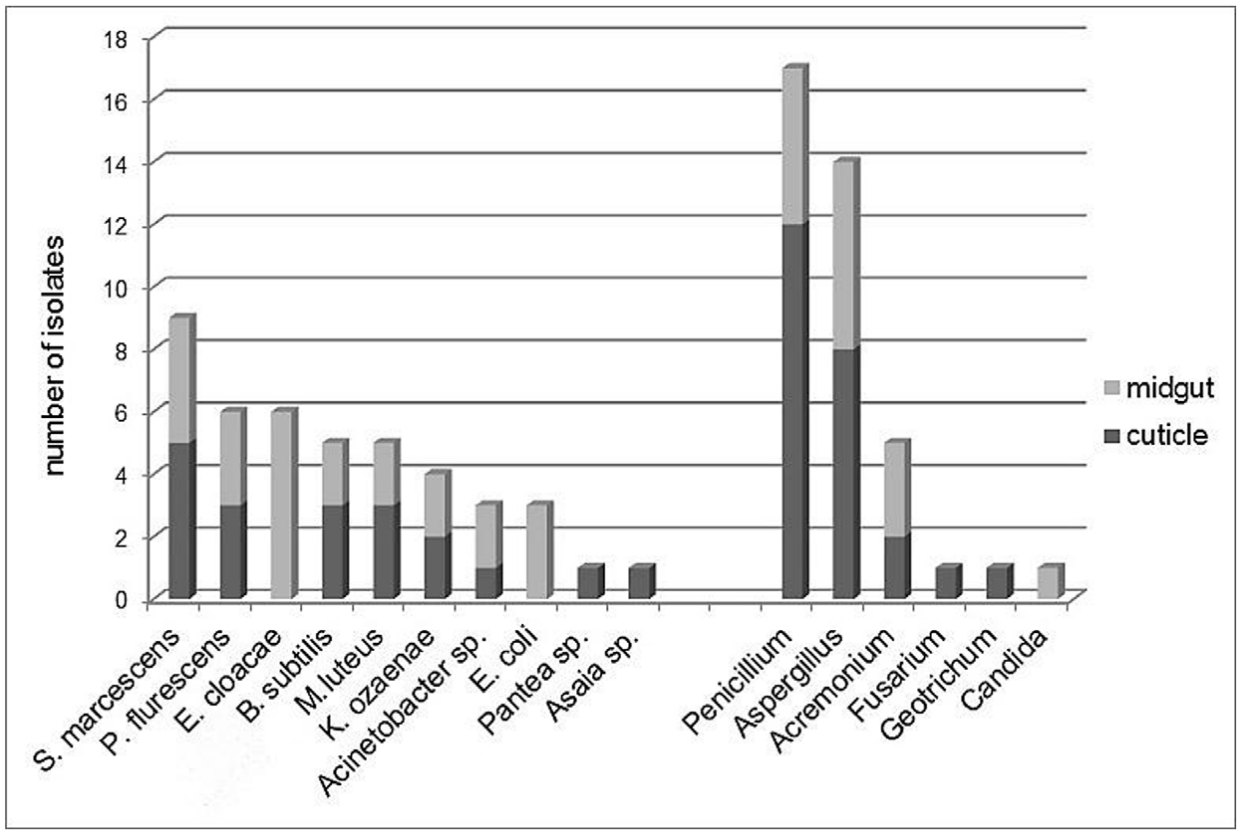
Origin and frequency of isolation of the different microorganisms.

A total of 39 fungal strains belonging to six genera and 43 bacterial strains were isolated from the 35 processed Phlebotomine sand flies. *Penicillium* (17 isolates) and *Aspergillus* (14 isolates) occurred more frequently in sand flies than the other genera that were present, i.e. *Acremonium* (5 isolates), and *Fusarium* (1 isolate), *Geotrichum* (1 isolate) and *Candida* (1 isolate) (Figure 5).

The *Penicillium* genera isolated in the present study showed different morphological features, suggesting that three different species were present but we were unable to identify at the species level. Consequently, we called them *Penicillium* sp.1, *Penicillium* sp.2 and *Penicillium* sp.3 respectively.

The bacterial isolates corresponded to ten bacterial taxa: *Serratia marcescens* (9 isolates), *Pseudomonas fluorescens* (6 isolates), *Klebsiella ozaenae* (4 isolates), *Acinetobacter* sp. (3 isolates), *Bacillus subtilis* (5 isolates) and *Micrococcus luteus* (5 isolates) isolated from both cuticle and midgut. *Enterobacter cloacae* (6 isolates) and *Escherichia coli* (3 isolates) as well as *Candida albicans* were isolated only from midgut whereas some other were specifically isolated from the cuticle like the bacteria belonging to *Asaia* sp. (1 isolate) and *Pantea* sp. (1 isolate) as well as the fungi including *Geotrichum* and *Fusarium* (see Figure 5).

## Discussion

In Phlebotomine sand flies, studies carried out on the gut flora of the wild or laboratory reared *Lutzomyia longipalpis*, *P. papatasi*, *P. tobbi*, *P. argentipes*, *P. duboscqi* and *Sergentomyia* spp. have demonstrated the presence of huge diversity of bacterial strains that belong to the genera *Acinetobacter*, *Bacillus, Brevibacterium*, *Burkholderia, Cellulomonas, Chloroflexi, Citrobacter, Enterobacter, Escherichia, Flavimonas, Gordonia, Klebsiella, Maltophila, Microbacterium*, *Micrococcus*, *Morganella, Ochrobactrum, Oligella, Pantoea, Pseudomonas, Serratia, Shigella, Sphingobacterium, Staphylococcus, Stenotrophomonas, Streptococcus* and *Weeksella* [14], [15], [16], [17], [19], [20], [25].In agreement with these investigations, bacteria belonging to the genera *Acinetobacter*, *Bacillus, Enterobacter*, *Escherichia*, *Klebsiella, Micrococcus*, *Pseudomonas* and *Serratia* were isolated from our samples. *E. cloacae* being the most common bacteria isolated from the sand flies’ gut. This result is in agreement with other studies carried out on sand flies [15], [16], [17], [19], [20], [26,]. *Asaia* sp. that has never been previously reported from sand flies has been isolated in the present study. We also report the isolation of *K. ozaenae* from both cuticle and midgut of four females. This bacterium was previously reported once from the midgut of *Lutzomyia longipalpis*, the vector of visceral leishmaniasis in the Americas [1].

Several studies have reported a higher prevalence of Gram-negative bacteria than Gram-positive ones in the gut of different vector insects [19], [20], [25], [27]. The lower prevalence of Gram-positive bacteria as compared to Gram-negative ones is due to antimicrobial activity against *M. luteus* and *B. subtilis* [20], [28] which make them less susceptible to colonization by Gram-positive bacteria. This is in agreement with our observation that the majority of the bacterial strains isolated in the present study were Gram-negative bacteria (76%) mainly *Enterobacter*, *Serratia* and *Pseudomonas*. Some studies have investigated a correlation between the presence of midgut bacteria and the development of parasites in flies. A high concentration of bacteria (mainly Gram-negative) in the midgut of mosquitoes as well as sand flies was reported to either completely or partly influence the development of parasites [2], [20], [29], [30]. In mosquitoes, a wide range of bacterial strains such as *Serratia*, *Klebsiella*, *Acinetobacter*, *Micrococcus*, *Escherichia, Enterobacter*, *Micrococcus, Pseudomonas, Staphylococcus* were pointed out as being symbionts in gut flora [31], [32], [33]. There is general agreement on the important role played by the gut microbial flora on the development of pathogens in the midgut of the insect. *Wolbachia* are common and widespread cytoplasmically inherited bacteria, found in reproductive tissues of arthropods, including Phlebotomine sand flies [34], [35]. Its interactions with its hosts are often complex and have evolved to be symbiotic rather than parasitic [34], [35], [36]. The lack of *Wolbachia* isolated in the present study might be due to the isolation and characterization methodology that we have used.

Several studies have reported the inhibitory activity of Gram-negative bacteria on the development of parasites in the mosquitoes’ gut [28], [31], [33], [37], [38], [39]. In sand flies, a study [19] reported a high prevalence of microbial infection in the digestive tract of laboratory reared *Phlebotomus papatasi* females and hypothesized they could have a negative effect on *Leishmania* transmission in endemic areas. However, these studies have not clearly identified the causal mechanisms explaining the impact of microbial infection on the intravectorial development of *Leishmania*. It is as yet impossible to know whether the microflora of sand flies could affect the development of *Leishmania* or not. Because of the low prevalence of *Leishmania* spp. in the sand flies’ gut, we could not explore this link in the present study but future studies on colonized species should be done to clarify the relationship. Interestingly, promastigotes of *Leishmania* in culture grow with difficulty when competing with bacteria. In the same way, bacteria can interfere with the development of promastigotes in the digestive tract of sand flies probably by competing for nutrients and reducing the pH [14]. In nature, despite the probable well-balanced associations between some bacteria and sand flies, there could be natural selective pressure involving some species of bacteria, *Leishmania* and their vectors.

Only a few publications that have aimed to identify fungal diversity are available and the majority of these are from work on mosquitoes [39], [40] only one [19] has been carried out on Phlebotomine sand flies. That study showed that only *Aspergillus sclerotiorum* and *Saccharomyces cerevisiae* were present in sand flies. In our study, we did not isolate and identify these two species, but we isolated and identified other species belonging to the *Aspergillus* genera (*A. flavus*, *A. fumigatus*, *A. nidulans*, *A. terreus*), *Penicillium*, *Geotrichum*, *Fusarium, Acremonium* and *Candida*. It was not possible for us to explain the role of these fungi which we isolated from the sand fly cuticle and/or midgut or reach any conclusion about any potential pathogenic effect or interaction with *Leishmania.* However, Adler and Theodor [41] and Schlein et al. [19] have proposed that female *P. papatasi* infected by fungal strains were significantly more resistant to *Leishmania major* infection.

The eggs of Phlebotomine sand flies are laid in soil that is rich in organic matter and the larvae pass through four instars in the soil before pupation and adult emergence. Consequently, the local soil environment and animal stools may play an important role in the colonization capacity of the sand flies, with microorganisms geographically specific encountered at the oviposition sites or during sugar meal feeding [42].

A large number of soil and environmental strains including *Acinetobacter* sp., *Asaia* sp., *B. subtilis, M. luteus, P. fluorescens, Pantoea* sp. and *S. marcescens* and also intestinal strains such as *E. cloacae, E. coli* and *K. ozaenae* were identified in the present study. These bacterial species belong to aerobic and facultative anaerobic Gram negative bacteria (*Acinetobacter* sp., *Asaia* sp., *E. cloacae*, *E. coli, K. ozaenae, Pantoea* sp., *P. fluorescens* and *S. marcescens*) and Gram positive bacteria (*B. subtilis* and *M. luteus*). According to the ecology of the bacteria and fungi isolated in the present study, it is probable that *E. coli*, *Enterobacter*, *Klebsiella*, *Pantoea*, *S. marcescens* or *C. albicans*, which all are intestinal microorganisms, have contaminated the sand flies during their larval stages. The telluric species like *Acinetobacter*, *Asaia*, *Bacillus subtilis*, *M. luteus* and *Pseudomonas* spp. could have contaminated the larvae, but also the adults in their resting places. The ubiquitous *Aspergillus* and *Penicillium* which are also telluric microorganisms, have probably contaminated adult sand flies.

According to our findings, the average number of counted bacterial strains in females with different physiological digestive stages has increased progressively from unfed to gravid females, like the prevalence of bacteria and fungi in the midgut as well as on the cuticle of female sand flies (Table 1). The same phenomenon has been also observed on mosquitoes [14], [20]. The authors of these studies suggested that the variation of the bacterial species in mosquitoes gut increased during the 24–48 hours following the blood feeding. This hypothesis could be applied to the Phlebotomine sand flies that we processed in this study. Another explanation could be that the life span of females is longer than that of males [35], [43]. Therefore, it seems probable that females may encounter more fungal and bacterial contaminants during their life span.

The present paper constitutes an interesting pilot study with new findings on the isolation of bacteria and fungi on the cuticle and in the gut of sand flies. It also suggests interesting trends like an increasing number of bacterial strains and colonies depending on the physiological stage of Phlebotomine sand flies. However, these data have to be confirmed in the future by further studies carried out on more specimens.
